# When Is It Appropriate to Take Off the Mask? Signaling Pathways That Regulate ß(1,3)-Glucan Exposure in *Candida albicans*

**DOI:** 10.3389/ffunb.2022.842501

**Published:** 2022-03-09

**Authors:** Tian Chen, Andrew S. Wagner, Todd B. Reynolds

**Affiliations:** ^1^Department of Pathogenic Biology, School of Biomedical Sciences, Shandong University, Jinan, China; ^2^Department of Microbiology, University of Tennessee, Knoxville, Knoxville, TN, United States

**Keywords:** unmasking, *Candida*, cell wall, signal transduction, MAP kinase, -glucan

## Abstract

*Candida* spp. are an important source of systemic and mucosal infections in immune compromised populations. However, drug resistance or toxicity has put limits on the efficacy of current antifungals. The *C. albicans* cell wall is considered a good therapeutic target due to its roles in viability and fungal pathogenicity. One potential method for improving antifungal strategies could be to enhance the detection of fungal cell wall antigens by host immune cells. (1,3)-glucan, which is an important component of fungal cell walls, is a highly immunogenic epitope. Consequently, multiple host pattern recognition receptors, such as dectin-1, complement receptor 3 (CR3), and the ephrin type A receptor A (EphA2) are capable of recognizing exposed (unmasked) (1,3)-glucan moieties on the cell surface to initiate an anti-fungal immune response. However, (1,3)-glucan is normally covered (masked) by a layer of glycosylated proteins on the outer surface of the cell wall, hiding it from immune detection. In order to better understand possible mechanisms of unmasking (1,3)-glucan, we must develop a deeper comprehension of the pathways driving this phenotype. In this review, we describe the medical importance of (1,3)-glucan exposure in anti-fungal immunity, and highlight environmental stimuli and stressors encountered within the host that are capable of inducing changes in the levels of surface exposed (1,3)-glucan. Furthermore, particular focus is placed on how signal transduction cascades regulate changes in (1,3)-glucan exposure, as understanding the role that these pathways have in mediating this phenotype will be critical for future therapeutic development.

## Introduction

*Candida* species are the most common human fungal pathogens and are also ranked as the fourth most frequent cause of hospital-acquired bloodstream infections, with up to 40% mortality in epidemiological studies (Wisplinghoff et al., 2004; Horn et al., 2009). *Candida* species colonize the human gastrointestinal tract and skin asymptomatically in many immunocompetent individuals. However, under certain conditions, *Candida* species can cause mucosal or systemic infections. Risk factors for each infection type vary, with risk factors for systemic infections including central venous catheter implants, major surgeries such as organ transplants, neutropenia, and cancer therapy (Karabinis et al., 1988; Wiley et al., 1990; Bertagnolio et al., 2004; Morrell et al., 2005). Risk factors for oropharyngeal mucosal infections include dentures, premature birth, and HIV infection, while hormone replacement therapy and diabetes are associated with increased risk of acquiring vulvovaginal infections (Pienaar et al., 2010; Pankhurst, 2013; Peterson et al., 2015). Current anti-fungal drugs for systemic *Candida* infections include three major classes—azoles, polyenes, and echinocandins, each of which target a specific aspect of the cell envelope (plasma membrane-cell wall complex) (Eggimann et al., 2003; Bustamante, 2005). However, a combination of drug toxicity, drug resistance and poor oral availability (Ostrosky-Zeichner et al., 2003; Perlin, 2015; Whaley et al., 2016) have limited the efficacy of these options.

The majority of life-threatening fungal infections are opportunistic in nature, and a novel approach that may be necessary to complement current antifungals will be to simultaneously improve host immune efficacy. This can include cytokine therapy and other adjunctive therapeutic approaches (Casadevall and Pirofski, 2001; Netea et al., 2008). A related strategy to improve adjunctive therapy that specifically targets the fungi themselves is to make fungal cells more recognizable to the host immune effector cells. A better understanding of the interaction of *Candida* species with their cognate host receptors, particularly for how different cell wall components are recognized by their receptors on immune cells, will provide new insights that will facilitate such an approach, while also improving our understanding of the process of fungal pathogenesis.

The recognition of fungal pathogens is the first step in coordination of the host's response to fungal infections, and appears to play an important role in shaping fungal colonization of the host by commensal fungi like the *Candida* spp. (Gantner et al., 2005; Taylor et al., 2007; Sem et al., 2016). Fungal cell wall composition and architecture are therefore critical in modulating the host immune response. Consequently, *Candida* spp. have developed strategies for sensing environmental signals encountered within the host in order to regulate the exposure of immunogenic epitopes located within their cell wall. This review will summarize the various conditions leading to the modification of cell wall structure and epitope exposure in *C. albicans*, including the internal regulatory pathways that are responsible for sensing and inducing these changes, and will highlight their immune-modulatory significance.

## Immunogenic Properties of the C. Albicans Cell Wall

**Figure 1:**
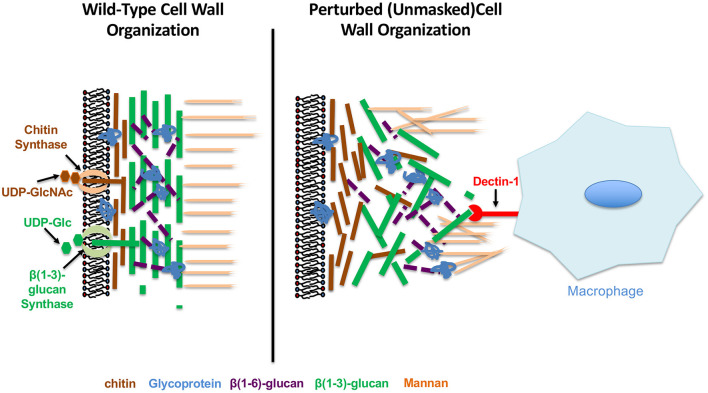
Wild-type and perturbed *Candida albicans* cell wall organization.

The *C. albicans* cell wall is a firm, but dynamic structure that is essential for fungal viability since it serves as a tough, but malleable barrier that sustains cell shape and prevents osmotic lysis (Chaffin, 2008; Free, 2013). The cell wall of *C. albicans* is a layered structure, consisting of an outer mannan layer that covers a central layer of the core structural polysaccharides (1,3)-glucan and (1,6)-glucan, as well as a basal chitin layer (Figure 1). The structural synthesis of the cell wall and each of its cognate components has been reviewed extensively and will not be discussed here (Chaffin, 2008; Hall and Gow, 2013; Gow et al., 2017; Garcia-Rubio et al., 2019; Ruiz-Herrera and Ortiz-Castellanos, 2019). However, each individual component of the *C. albicans* cell wall has been shown to have immunomodulatory properties, and therefore highlights the significance of this structure in mediating host-pathogen interactions.

### ß(1,3)-Glucan

(1,3)-glucan accounts for ~40% of the total mass of the *C. albicans* cell wall (Gow et al., 2007), and has been found to induce a strong pro-inflammatory immune response upon recognition by host pattern recognition receptors (PRRs) such as dectin-1 (Brown and Gordon, 2001; Brown et al., 2002; Taylor et al., 2002; Gow et al., 2007; Kennedy et al., 2007; Cohen-Kedar et al., 2014), complement receptor 3 (CR3) (Van Bruggen et al., 2009; Li et al., 2011) and the ephrin type A receptor 2 (EphA2) (Swidergall et al., 2018, 2019). As a central component of the cell wall, and as a consequence of the pro-inflammatory nature of this epitope, (1,3)-glucan exposure (referred to as unmasking) is highly regulated to ensure successful host immune system evasion during infection. This is highlighted by virulence defects during systemic infection with mutants that have increased (1,3)-glucan unmasking, such as deletions in the phosphatidylserine synthase gene *CHO1* (Chen et al., 2010), the glycosyltransferase gene *KRE5* (Herrero et al., 2004), the yeast cell wall protein *YWP1* (Yang et al., 2020) and the exo-1,3 and endo-1,3-glucanses *XOG1* and *ENG1*, respectively (Childers et al., 2020; Yang et al., 2020). Additionally, loss of host (1,3)-glucan PRRs has been shown to increase disease severity during both systemic infection (in dectin1^−/−^ and CR3^−/−^ mice) (Taylor et al., 2007; Li et al., 2011; Thompson et al., 2019) and mucosal infections (in dectin1^−/−^ and epha2^−/−^ mice) (Gales et al., 2010; Carvalho et al., 2012; Swidergall et al., 2018, 2019), further highlighting the importance of (1,3)-glucan recognition in disease control. However, it is important to note that dectin-1 mediated clearance was found to be strain specific to *C. albicans* (Saijo et al., 2007; Marakalala et al., 2013), and appears to be impacted by basal chitin levels within the cell wall of different isolates. Therefore, cell wall architecture is an important mediator in facilitating the dectin-1 immune response.

### Mannosylated Cell Wall Components

The outer layer of the fungal cell wall is largely composed of heavily glycosylated proteins and lipids, and serves as the first interface between fungal cells and the host. Mannan is the most prevalent carbohydrate decorating these outer cell wall proteins, accounting for ~80–90% of all attached sugar moieties (Netea et al., 2015). As a consequence of the high abundance of mannosylated cell wall structures and their exposure to the host environment, there are several PRRs capable of recognizing mannosylated residues decorating the outer cell wall. These include dectin-2 (α-mannan) (Mcgreal et al., 2006; Saijo et al., 2010), dendritic cell-specific intracellular adhesion molecule-3-grabbing non-integrin (DC-SIGN) (*N-*mannan) (Cambi et al., 2008), mannose receptor (MR) (*N-*mannan) (Netea et al., 2006; Cambi et al., 2008), galectin-3 [(1,2)-mannosides] (Fradin et al., 2000; Jouault et al., 2006; Kohatsu et al., 2006), and the Toll-like receptors TLR4 (*O-*mannan) (Netea et al., 2006) and TLR2 (phospholipomannan) (Jouault et al., 2003).

The impact that mannose-binding PRRs have on disease progression has been studied *in vitro* and *in vivo*, and each receptor has been shown to impact disease either directly or in combination with additional host PRRs. For example, dectin-2^−/−^ mice systemically infected with *C. albicans* display reduced survival and increased fungal burden (Saijo et al., 2010), and this phenotype is further exacerbated during systemic infection in dectin-1 and dectin-2 double knockout mice (dectin-1^−/−^dectin-2^−/−^) (Thompson et al., 2019), suggesting a synergistic role for these receptors in mediating disease control. Similarly, galectin-3^−/−^ mice are more susceptible to systemic infection with *C. albicans* and display higher levels of kidney fungal burden than immunocompetent wild-type mice (Linden et al., 2013). This receptor is also required for full activation of TNFα expression when dectin-1 is activated by (1,3)-glucan *in vitro* (Esteban et al., 2011), further highlighting the interactions between host PRRs. TLR2 receptors have also been shown to synergize with dectin-1 signaling in responses to zymosan [autoclaved yeast cells that expose (1,3)-glucan] by increasing pro-inflammatory cytokine production *in vitro*, in spite of the fact that TLR2 alone does not directly recognize zymosan nor activate immune responses sufficiently when stimulated by zymosan preparations (Gantner et al., 2003). However, *in vivo* studies on the impact that TLR2 has during systemic infection have been confounding in nature, with contradictory reports demonstrating increased survival during systemic infection with *C. albicans* in TLR2^−/−^ mice (Netea et al., 2004), while other reports have demonstrated reduced survival during systemic infection and increased dissemination during intraperitoneal infection in TLR2^−/−^ mice (Villamon et al., 2004; Tessarolli et al., 2010). Systemic infection in TLR4^−/−^ mice has also been shown to increase susceptibility to *C. albicans* in a strain specific manner, and is marked by increased fungal burden and impaired neutrophil recruitment in isolates recognized by TLR4 (Netea et al., 2002, 2010). Finally, intraperitoneal infection in MR^−/−^ mice does not appear to impact survival rates when compared to infected wild-type mice (Lee et al., 2003). Yet, loss of MR does have a role in regulating fungal burden, as increased fungal burden in the lungs and brain at 7 days post-infection (d.p.i.) and in the spleen and lungs at 21 d.p.i. has been observed in MR^−/−^ mice.

In addition to their ability to stimulate the immune system directly, mannosylated proteins in the outer cell wall have also been proposed to serve a protective role for *C. albicans* during infection by covering (masking) immunogenic (1,3)-glucan epitopes within the central layer of the cell wall. This is supported by the observation that mutations in genes of the *MNN2* α(1,2)-mannosyltransferase family, components of the *N-*glycosylation pathway, significantly reduce mannan levels in the outer cell wall, while also increasing the levels of exposed (1,3)-glucan (Graus et al., 2018). Likewise, loss of glycosylphosphatidylinositol (GPI)-anchored proteins, a major constituent of the outer cell wall (Chaffin, 2008; Gow et al., 2017), have also been shown to increase -glucan exposure. Genetic deletion of *GPI7*, an essential protein in GPI anchor synthesis, blocks the decoration of the second mannose in the glycan with phosphoethanolamine and subsequently unmasks (1,3)-glucan (Shen et al., 2015). Additionally, chemical inhibition of GPI anchor synthesis with gepinacin, which inhibits the activity of a critical acyltransferase Gwt1, or with the 2-aminonicitinomide derivative 11g, increased (1,3)-glucan exposure in the cell wall and resulted in increased cytokine production from murine macrophages (Umemura et al., 2003; Mclellan et al., 2012; Ni et al., 2017; Huang et al., 2020). In the case of 11g treatment, this also corresponded with increased survival and reduced kidney fungal burden during systemic infection in mice (Huang et al., 2020).

### Chitin

As the most basal component of the *C. albicans* cell wall, chitin represents ~10% of the total cell wall weight (Gow et al., 2017). The direct role that chitin plays as an immunogen during *C. albicans* infection is still largely unknown. However, (Wagener et al., 2014) have shown that purified chitin particles are capable of eliciting both a pro-inflammatory response (TNFα secretion) by murine macrophages exposed to high concentrations of purified chitin and an anti-inflammatory response (IL-10 secretion) at low concentrations, demonstrating a concentration dependent impact for chitin on immune stimulation (Wagener et al., 2014). This anti-inflammatory response was also found to be dependent on the presence of the intracellular nucleotide binding oligomerization domain containing 2 (NOD2) and TLR9 receptors, implicating these host PRRs in the chitin induced IL-10 response.

In addition to its ability to regulate immune system activation, chitin levels have also been indirectly associated with (1,3)-glucan exposure. Although it is usually considered to be more basal, large additions of chitin seem to cause substantial unmasking. This has been observed for regions damaged on hyphae by neutrophils, cell wall changes induced by caspofungin treatment, clinical isolates from symptomatic vulvovaginal candidiasis patients, and mutants like a hyperactive *STE11*^Δ* N*467^ mutant (a MAP3K of the Cek1 MAPK pathway) and a *cho1*Δ*/*Δ mutant (Wheeler et al., 2008; Davis et al., 2014; Hopke et al., 2016; Pericolini et al., 2018; Wagner et al., 2021). This latter mutant is of particular interest, as the cell wall in the *cho1*Δ*/*Δ mutant is accompanied by a loss of the smooth outer structure of the cell and an increase in surface rigidity (Hasim et al., 2017). In addition to the possibility of unmasking being caused by loss of cell wall mannans, another model to explain unmasking is that more basal polymers like chitin may be deposited in such a way that they disrupt the architectural substructure of the cell wall, and cause significant exposure of (1,3)-glucan (Figure 1).

## Environmental Signals That Decrease C. Albicans ß(1,3)-Glucan Exposure

As a consequence of the immunogenic nature of fungal cell wall epitopes, fungal pathogens have developed mechanisms for immune avoidance that involve concealing (masking) (1,3)-glucan (Wheeler and Fink, 2006; Rappleye et al., 2007; Wheeler et al., 2008; Gravelat et al., 2013; Gow et al., 2017). In *C. albicans*, (1,3)-glucan is buried underneath an outer layer of glycosylated proteins in the cell wall in a phenomenon referred to as masking (Wheeler and Fink, 2006; Wheeler et al., 2008; Gow et al., 2017). The process of concealing (1,3)-glucan is largely impacted by exogenous signals encountered by fungal cells, and represents an adaptive feature deployed by *C. albicans* in response to the diverse niches that it may encounter.

### Hypoxia-Induced Masking

Low oxygen abundance is a common stressor encountered by *C. albicans* in both its commensal and pathogenic states. As a commensal organism, hypoxia toleration must be achieved to permit colonization of the lower gastrointestinal tract (He et al., 1999; Rosenbach et al., 2010) and is regularly encountered once pathogenesis ensues in inflamed and necrotic tissue (Eltzschig and Carmeliet, 2011; Grahl et al., 2012). Consequently, *C. albicans* induces robust changes in both its metabolism and cell wall organization/composition to adapt to hypoxic conditions (Setiadi et al., 2006; Sosinska et al., 2008; Burgain et al., 2020). Pradhan et al. (2018, 2019) have observed that one such change induced by hypoxia is a reduction in the levels of exposed (1,3)-glucan moieties in the cell wall. This is accompanied by reduced phagocytosis of hypoxia exposed *C. albicans* cells by murine macrophages and decreased cytokine production (specifically IL-10 and CCL5) by human peripheral blood mononuclear cells (hPBMCs).

**Figure 2:**
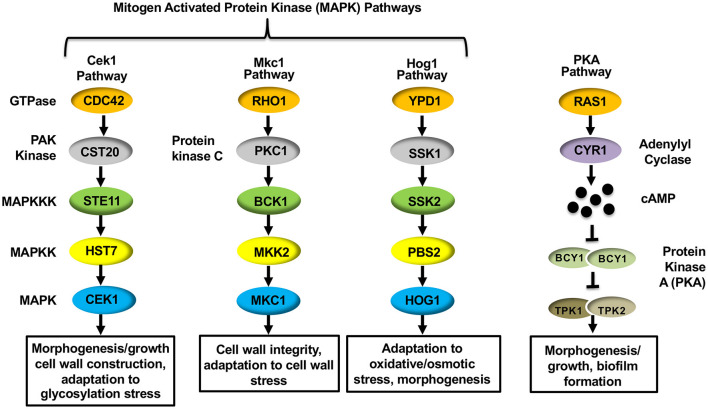
Signal transduction pathways within *Candida albicans* that regulate ß(1,3)-glucan exposure.

The mechanism driving hypoxia-induced masking was found to depend on proper mitochondrial functioning, as mutations in *C. albicans* genes that are important for mitochondrial respiration (*GOA1* and *UPC2*) and reactive oxygen species (ROS) processing (specifically by the alternative oxidase *AOX1* and the mitochondrial localized superoxide dismutase *SOD1*) impacted (1,3)-glucan masking (Pradhan et al., 2018). Furthermore, the cAMP-Protein Kinase A (cAMP-PKA) pathway was also necessary to reduce (1,3)-glucan exposure. The cAMP-PKA pathway has been implicated in regulating yeast-to-hyphae and white-to-opaque morphogenic switching, as well as virulence of *C. albicans* (Sonneborn et al., 2000; Bockmuhl et al., 2001; Rocha et al., 2001; Ding et al., 2016). The canonical pathway consists of two upstream GTPases (Ras1 and Ras2), an adenylyl cyclase (Cyr1) that is necessary for producing cAMP, and the downstream protein kinase A (PKA) that is composed of catalytic (Tpk1 and Tpk2) and regulatory (Bcy1) subunits that respond to cAMP production (Figure 2) (Huang et al., 2019). Within the cAMP-PKA pathway, deletions of *CYR1* or simultaneous deletions of both PKA catalytic subunits (*tpk1*Δ*/*Δ*tpk2*Δ*/*Δ) blocked masking caused by hypoxia exposure (Pradhan et al., 2018). The cAMP-PKA pathway was proposed to be regulated by proper ROS processing to hydrogen peroxide within the mitochondria, thus linking these processes together.

### Lactate-Induced Masking

In addition to changes in oxygen levels within different niches of the host, the major carbon sources within different anatomical locations also vary. For example, glucose levels are high in the blood and relatively low in the lower gastrointestinal tract (Barelle et al., 2006), where alternative carbon sources such as short chain fatty acids and lactic acid are more readily present (Yamaguchi et al., 2005; Rios-Covian et al., 2016). In order to adapt to these diverse conditions, *C. albicans* induces metabolic changes to better utilize the exogenous carbon sources in its immediate environment (Rubin-Bejerano et al., 2003; Vieira et al., 2010; Miramon and Lorenz, 2017). Carbon source availability has also been shown to impact cell wall architecture and epitope exposure in a carbon-source dependent manner. Namely, exposure to _L_-lactate or glycerol have been found to induce (1,3)-glucan masking in *C. albicans* when compared to glucose grown cells (the most prominently used carbon source for *in vitro Candida* growth) (Ballou et al., 2016; Pradhan et al., 2019), while exposure to the short chain fatty acids acetate or butyrate inversely increased (1,3)-glucan unmasking (Pradhan et al., 2019). With respect to _L_-lactate exposed cells, epitope masking correlated with decreased production of pro-inflammatory cytokines, including TNFα and MIP1, by murine macrophages *in vitro* as well as reduced phagocytosis (Ene et al., 2013; Ballou et al., 2016). This further highlights the immunomodulatory impact that (1,3)-glucan exposure has on virulence-related phenotypes.

The mechanism driving _L_-lactate-induced masking has been explored, and two signal transduction pathways have been found to be essential in this response. Ballou et al. (2016) have shown that masking induced by _L_-lactate is mediated *via* a signal transduction pathway that requires the G-protein coupled receptor Gpr1 and the downstream transcription factor Crz1. Pradhan et al. (2019) then elaborated on this work and showed that the cAMP-PKA pathway is also needed to induce masking in response to lactate exposure, as deletions of both *CYR1* and *TPK1* both blocked (1,3)-glucan masking. Gpr1 has also been shown to interact with the cAMP-PKA pathway to mediate hyphal formation (Miwa et al., 2004; Maidan et al., 2005). In response to _L_-lactate-driven masking, Gpr1 may serve as a receptor. The observation that the cAMP-PKA pathway is necessary for _L_-lactate-induced masking, as well as hypoxia-driven masking (see section above) (Pradhan et al., 2018), implicates this pathway as a conserved regulator for (1,3)-glucan masking. However, Crz1 is a well-known effector of calcineurin when stimulated by exogenous Ca^2+^ (Karababa et al., 2006). As deletion of the structural subunits of calcineurin did not impact _L_-lactate-induced masking (Ballou et al., 2016), the observation that Crz1 mediates this phenotype represents a non-canonical pathway for Crz1 activation. Childers et al. (2020) reported that Crz1 and Gpr1 are both needed to induce expression of the exo-1,3--glucanase Xog1 in response to _L_-lactate. Xog1 cleaves exposed (1,3)-glucan moieties in the cell wall to cause masking, and thus represents an effector gene mediated by this pathway in response to lactate exposure. However, Crz1 regulation of this pathway may not be entirely direct, as it regulates expression of the transcription factor *ACE2* as well. *ACE2* is also required for lactate-induced masking (Ballou et al., 2016), but a direct role of Ace2 in regulating *XOG1* expression in response to _L_-lactate has not been assessed.

### Iron Limitation-Induced Masking

Nutrient sequestration (in a process referred to as nutritional immunity) is an additional host stressor that *C. albicans* must overcome in order to successfully induce disease (Hood and Skaar, 2012; Crawford and Wilson, 2015). One such stressor of nutritional immunity that is capable of inducing metabolic and architectural changes in the cell wall of *C. albicans* is a reduction of trace metals in the surrounding environment (Chen et al., 2011; Li et al., 2015; Malavia et al., 2017). Pradhan et al. (2019) have reported that specific depletion of copper and iron were sufficient to induce changes in the cell wall of *C. albicans* and induce masking of immunogenic (1,3)-glucan moieties, while the loss of zinc and manganese inversely increase (1,3)-glucan exposure. With respect to iron limitation, which induced the strongest change in (1,3)-glucan exposure levels of these metals, the observed masking was also associated with decreased phagocytosis by bone marrow derived macrophages (BMDMs) and reduced secretion of the pro-inflammatory cytokines TNFα, IL-6, and MIP-s1α by hPBMCs. Iron-induced masking was found to be regulated by two parallel signal transduction pathways, one that is again mediated by PKA and another that consists of the iron transceptor Ftr1 and the iron-responsive transcription factor Sfr1. However, it is important to note that iron-depletion-induced masking was not dependent on Cyr1, as was observed for hypoxia and _L_-lactate induced masking. Thus, although PKA is involved in iron depletion-induced, hypoxia-induced, and lactate-induced masking, the mechanism by which it is activated in response to iron-reduction is non-canonical.

## Environmental Signals That Increase C. Albicans ß(1,3)-Glucan Exposure (Unmasking)

In addition to host stimuli that induce masking of (1,3)-glucan, fungal pathogens also encounter stressors that alter the normal architecture of the cell wall to further increase immunogenic epitope exposure (Figure 1). These changes come in multiple forms, and can be induced by both environmental signals sensed by *C. albicans*, or mechanical and physical perturbances encountered *in vivo* that impact cell wall integrity and synthesis.

### Acidic pH

Changes in host pH levels vary drastically between the various niches that *C. albicans* can inhabit. This is apparent in the strongly acidic pH's encountered in the stomach and vaginal lumen (pH ~2 and 4–5, respectively), as opposed to the range of pH's (~4–7.4 in the gastrointestinal tract) in the lower gastrointestinal tract (Fallingborg, 1999; Vylkova et al., 2011; Gunther et al., 2014). In response to alterations in environmental pH, Sherrington et al. (2017) have revealed that *C. albicans* adaptation to an acidic pH of 4.0 or below promotes cell wall remodeling, increases exposure of both chitin and (1,3)-glucan, and reduces thickness of the outer mannan layer. These changes also correspond with enhanced immune recognition, leading to increased phagocytosis by macrophages and neutrophils and increased pro-inflammatory cytokine production (TNFα, IL-6, IL-1) by PBMCs.

The mechanism by which (1,3)-glucan exposure is induced by acidic pH remains to be fully defined, but chitin exposure was found to be regulated by the transcription factors Bcr1 and Rim101 and was dependent on the repression of the fungal chitinase Cht2 (Sherrington et al., 2017). In this model, *CHT2* expression is promoted in alkaline environments by both Bcr1 and Rim101, but in acidic environments the Rim101 pathway is repressed and *CHT2* expression is decreased. This change is proposed to lead to longer chitin fibrils due to decreased microfibril processing by Cht2, thus impairing efficient remodeling of the inner cell wall layer and enhancing exposure of chitin at the cell surface. Interestingly, after about 2–4 h at pH 4.0, glucan and chitin are remasked (Cottier et al., 2019). The process of -glucan remasking, but not remasking of chitin, can be blocked by treatment with the *C.albicans* quorum-sensing molecule farnesol, suggesting a role for the farnesol response pathway in mediating these changes. Moreover, loss of the transcription factor Efg1 reduced the levels of exposed chitin, but not of -glucan. Loss of *EFG1* was found to derepress *CHT2* expression at an acidic pH of 4, which was proposed to be the mechanism driving these changes.

### Host Immune Response-Induced Unmasking

During infection, unmasking can occur in response to the host environment. Wheeler et al. (2008) described a time-course of (1,3)-glucan exposure during disseminated infection in mice by *C. albicans*. The polymer is masked by the outer layer of glycoproteins at the beginning of infection, with ~20% of fungal cells showing exposed (1,3)-glucan foci at 16 h post-infection, and by 7 d.p.i. the percentage of cells showing unmasking increased to 80%. The exact mechanism by which unmasking occurs *in vivo* is not fully understood, but it has been observed that when *C. albicans* hyphae are exposed to neutrophils *in vitro*, that these immune cells damage the cell wall *via* neutrophil extracellular trap (NET) production and cause (1,3)-glucan unmasking (Hopke et al., 2016). Neutrophil-damage of hyphae results in cell wall remodeling, during which the outer layer of glycosylated proteins is damaged within seconds upon neutrophil attack, followed by chitin deposition 30 min post-insult and then β-glucan unmasking. This process was found to depend on the mitogen activated protein kinase (MAPK) Hog1, as *hog1*Δ/Δ hyphal cells failed to show unmasking upon NET exposure. Hog1 is a known regulator of morphogenesis, and has been extensively implicated in mediating stress responses to both osmotic and oxidative insults (Figure 2) (Alonso-Monge et al., 1999, 2001; Monge et al., 2006). However, a *hog1*Δ/Δ mutant does not exhibit any changes in (1,3)-glucan exposure during growth as yeast form cells in culture (Correia et al., 2019). Thus, this study highlights a stimulus-dependent role for Hog1 in mediating unmasking, and it is possible that this relationship holds true for some other MAPKs of *C. albicans* as well. In addition to inducing (1,3)-glucan exposure, the Hog1-mediated NET response also increased production of chitin *via* the chitin synthase Chs3, the major chitin synthase of *C. albicans* (Mio et al., 1996; Hopke et al., 2016). Although a causative role for chitin deposition in increasing (1,3)-glucan exposure has yet to be empirically demonstrated, the observation that chitin levels are increased following NET insult before (1,3)-glucan unmasking occurs further strengthens the correlative evidence that chitin plays a role in impacting -glucan epitope exposure.

### Drug-Induced Exposure

Three main classes of antifungals are currently used in the treatment of candidiasis (Eggimann et al., 2003; Bustamante, 2005) and the impact that each of them has on immunogenic epitope exposure has been studied both *in vitro* and *in vivo*. Amongst all antifungals, the impact that the echinocandin caspofungin has on (1,3)-glucan exposure in *C. albicans* is the most well-studied (Wheeler and Fink, 2006; Wheeler et al., 2008; Tams et al., 2020). Wheeler and Fink (2006) reported that sub-lethal dosages of caspofungin upset the intricate cell wall structure of *C. albicans* and causes (1,3)-glucan exposure without killing the fungi *in vitro*. This increased exposure in turn elicits more potent pro-inflammatory responses, including TNFα secretion, from macrophages through dectin-1 binding. Furthermore, caspofungin treatment in mice prior to systemic infection maintains the ability to induce unmasking of fungal cells, and therefore demonstrates that this phenotype is maintained *in vivo* (Wheeler et al., 2008). Amphotericin B, a member of the polyene drug class, has also been shown to induce unmasking upon exposure *in vitro* (Pradhan et al., 2019), although the impact that amphotericin-induced unmasking has on immune stimulation is thus far understudied. Treatment with fluconazole, a member of the azole drug class, has been found to induce unmasking upon exposure to yeast cells *in vitro* (Pradhan et al., 2019). However, unlike caspofungin, this phenotype did not translate *in vivo*, as fungal isolates from mice that had been previously treated with fluconazole prior to infection did not show a difference in -glucan exposure when compared to fungal cells from untreated mice (Wheeler et al., 2008). Thus, more work is needed to better understand the role that fluconazole may play in eliciting -glucan exposure in a clinical setting.

In an attempt to better understand how caspofungin induces cell wall changes in *C. albicans*, Badrane et al. (2012) showed that rapid responses at the plasma membrane in components related to the actin cytoskeleton also occurred in addition to caspofungin-induced cell wall rearrangements. This includes redistribution of both phosphatidylinositol-(4,5)-bisphosphate [PI (4,5) P_2_], a signaling phospholipid, and septins, which serve as scaffolds for cytokinesis events (Badrane et al., 2012). PI (4,5) P_2_ and septins are re-localized 5 min after exposure to caspofungin, which represents an early event in response to drug treatment, and further re-directs chitin and cell wall proteins to deposit at the site of co-localization (Badrane et al., 2012). This response might be important for membrane localized cell wall repair proteins like chitin synthases to fix the damage caused by (1,3)-glucan biosynthesis inhibition. In fact, chitin production increases in response to caspofungin treatment (Badrane et al., 2012; Davis et al., 2014; Hasim et al., 2017). As increased chitin levels have been correlated with increased (1,3)-glucan unmasking (see above), the septins may play an upstream role in unmasking as well (Wheeler et al., 2008; Davis et al., 2014; Hopke et al., 2016; Hasim et al., 2017; Pericolini et al., 2018; Valotteau et al., 2019; Wagner et al., 2021).

## MAPK Pathways Mediating ß(1,3)-Glucan Exposure

It is well-established that both canonical and non-canonical signaling pathways are responsible for inducing changes in the *C. albicans* cell wall in response to environmental stimuli, and that this in turn impacts the levels of ß(1,3)-glucan epitopes exposed to the host environment. In addition to stimulus-mediated activation of these pathways, direct interrogation of additional signal transduction pathways *via* genetic manipulation of their cognate components has also been found to mediate -glucan exposure. This is particularly apparent for genetic modifications within the mitogen activated protein kinase (MAPK) pathways of *C. albicans*, which have already been heavily implicated in a wide range of stimuli responses, including osmotic stress, oxidative stress, cell wall damage, α-factor pheromone signaling, and changes in glycosylation (Monge et al., 2006; Roman et al., 2009; Galan-Diez et al., 2010; Ramirez-Zavala et al., 2013; Cullen and Edgerton, 2016; Hopke et al., 2016; Scaduto et al., 2017).

MAPK pathways are well-conserved signaling cascades in eukaryotes, and are composed of a conserved module of three kinases: a MAP kinase kinase kinase (MAP3K), a MAP kinase kinase (MAP2K) and a MAP kinase (MAPK) (Gonzalez-Rubio et al., 2019). Activation of the MAP3K induces a phosphorelay system, in which it phosphorylates (activates) the MAP2K, which then phosphorylates and activates the MAPK to transmit signaling. Once phosphorylated, the MAPK activates, *via* phosphorylation, the appropriate transcription factor to respond to the initial stimulus. *C. albicans* possess three such MAPK pathways: the Cek1, Mkc1, and Hog1 MAPK pathways (Figure 2). Information pertaining to the role of the Hog1 pathway in mediating -glucan unmasking has already been discussed (see “Host Immune Response-Induced Unmasking” above), but the impact that both the Cek1 and Mkc1 MAPK pathways have on regulating -glucan exposure in *C. albicans* will be elaborated below.

All three of these MAPKs are upregulated in response to caspofungin treatment (Munro et al., 2007; Roman et al., 2009), and both the Cek1 and Mkc1 pathways are upregulated in the *cho1*Δ/Δ mutant (Chen et al., 2019a,b). Since unmasking occurs in both of these conditions, the Cek1 and Mkc1 pathways were analyzed to determine if either could drive unmasking by being activated alone. This revealed that the Cek1 pathway had a clear ability to cause unmasking when activated in the absence of other stimuli. This has been a useful tool to study unmasking as it simplifies the system and is less pleiotropic than more complex stimuli like stress or the *cho1*Δ/Δ mutation. We describe the reported roles of the Cek1 and Mkc1 pathways in unmasking below.

### The Cek1 MAPK Pathway

The canonical Cek1 MAPK pathway consists of a core kinase cascade of Ste11-Hst7-Cek1/Cek2 (Figure 2) that regulates the activation of the downstream transcription factors Cph1 and Ace2 (Liu et al., 1994; Csank et al., 1998; Ramirez-Zavala et al., 2013; Van Wijlick et al., 2016; Wagner et al., 2021). This pathway is heavily involved in mediating cell wall organization, and has consequently been shown to play an active role in inducing morphogenic changes from yeast to hyphae (through Cph1) (Liu et al., 1994; Csank et al., 1998), from white to opaque phase in mating-competent *C. albicans* strains (through Cph1) (Ramirez-Zavala et al., 2013), mating signaling itself (Chen et al., 2002), and in responding to glycostructure damage in the cell wall (through Ace2) (Cantero et al., 2007; Roman et al., 2009; Van Wijlick et al., 2016). Due to the direct impact that the Cek1 MAPK pathway plays in regulating cell wall architecture, it is unsurprising that genetic alterations within the pathway also induce changes in immunogenic epitope exposure. The impact that deletion of *CEK1* itself has on unmasking has been the subject of conflicting reports, with some analyses showing increased unmasking upon deletion of *CEK1* (Galan-Diez et al., 2010; Correia et al., 2019) and others showing no impact on epitope exposure (Wagner et al., 2021). However, it has been shown that expression of a single hyperactive allele of the MAP3K *STE11*, in which the N-terminal 467 amino acid autoinhibitory domain has been deleted (*STE11*^Δ* N*467^*)*, causes hyperactivation of the downstream MAPK Cek1 and induces increased (1,3)-glucan unmasking, as well as increases in both total and exposed chitin levels within the cell wall (Chen et al., 2019a,b; Wagner et al., 2021). Hyperactive *STE11*^Δ* N*467^ expression in turn enhances survival in mice and attenuates fungal colonization in the kidneys, spleen and brain (Chen et al., 2019b; Wagner et al., 2021). Importantly, immunosuppression *via* cyclophosphamide treatment was able to restore both survival rates and kidney fungal burden to near wild-type levels (Wagner et al., 2021). Thus, the observed virulence defect is largely host immune system driven, and attenuation is likely the consequence of the increased epitope exposure observed during hyperactive *STE11*^Δ*N*467^ expression *in vitro*.

Ste11^Δ*N*467^-induced unmasking was further found to be mediated through the downstream transcription Cph1, as deletion of *CPH1* in a hyperactive *STE*11^Δ*N*467^ mutant restored (1,3)-glucan and chitin exposure to wild-type levels. *CPH1* deletion was also able to suppress the kidney fungal burden defect caused by the *STE11*^Δ*N*467^ allele in systemic infections (Wagner et al., 2021). Interestingly, *STE11*^Δ*N*467^ expression also induced activation of an unidentified parallel signaling pathway that is mediated through the putative cell wall sensor Dfi1. The Dfi1 protein is a 2 transmembrane protein that has been implicated in Cek1 activation and hyphal formation (Zucchi et al., 2010; Herwald et al., 2017). Although deletion of *DFI1* in a *STE11*^Δ*N*467^ mutant background partially suppressed unmasking toward wild-type levels, it did not appear to impact the activation status of Cek1 itself, suggesting that Dfi1 signaling is transmitted elsewhere. Thus, similar to what has been observed for hypoxia-induced (Pradhan et al., 2018) and iron-induced (Pradhan et al., 2019) unmasking, multiple pathways appear to function in unison to elicit the full levels of (1,3)-glucan exposure observed during *STE11*^Δ*N*467^ expression. Although the mechanism driving unmasking in a *STE11*^Δ*N*467^ mutant is unknown, RNA sequencing revealed an enrichment in structural cell wall genes (such as GPI-anchored proteins) and those involved in cell wall synthesis and repair (such as chitin synthase, chitinase, transglucosylase, and -glucanase genes) (Chen et al., 2019b), and it is likely that these changes work synergistically to increase (1,3)-glucan exposure during *STE11*^Δ*N*467^ expression.

Ace2, another transcription factor downstream of Cek1, has been shown to play an important role in sustaining cell wall structure in *C. albicans*. The *ace2*Δ/Δ mutant displays severe (1,3)-glucan exposure, and transcripts induced by Ace2 upregulation are thought to be partially responsible for (1,3)-glucan masking induced by lactate treatment (Liu et al., 1994; Ballou et al., 2016; Roman et al., 2016). This may be due to the ability of Ace2 to control the degradation of (1,3)-glucan (possibly *via* Xog1), or it may be the consequence of Ace2 regulation of either mannan or chitin synthesis within the cell. The expression of another glucanase, the exo-1,3--glucanase *ENG1*, has been implicated in regulating (1,3)-glucan exposure in yeast cells by directly degrading exposed (1,3)-glucan moieties in the outer cell wall (Yang et al., 2020). Loss of *ENG1* induces strong unmasking during exponential growth of yeast cells *in vitro* (Yang et al., 2020). *ENG1* has been shown to sit within the Ace2 regulon (Mulhern et al., 2006), and it may be possible that Ace2 regulates masking *via* this enzyme. However, Ace2-mediated changes in epitope exposure may be related to the role that Ace2 plays in controlling cell wall glycostructure (Van Wijlick et al., 2016). Damage of *N-*glycans is sensed by the cell wall proteins Msb2 and Sho1, and results in the activation of Cek1, which in turn activates cell wall repair activities mediated by Ace2 transcriptionally (Roman et al., 2009; Cantero and Ernst, 2011). In addition, Ace2 controls the expression of *O-*glycosylation genes in an isoform-specific manner (Van Wijlick et al., 2016). Under unstressed condition, Ace2 represses Pmt1 expression *via* the transcription factor Zcf21, and de-represses it in response to tunicamycin (Cantero and Ernst, 2011). Ace2 also has the ability to regulate basal chitin levels by influencing the expression of the chitinase gene *CHT3* (Kelly et al., 2004; Mulhern et al., 2006), which is the major chitinase responsible for homeostatic cell wall architecture and cell separation in yeast cells (Dunkler et al., 2005). Therefore, it may be possible that any one of these functions, and more than likely many of them, impact epitope exposure during *ACE2* deletion and in response to lactate exposure.

### The Mkc1 MAPK Pathway

In *C. albicans*, the Mkc1 MAPK cascade, consisting of Bck1-Mkk2-Mkc1 (Figure 2), is activated in response to exogenous cell wall stress, oxidative stimuli, antifungal drugs, and low-temperature shocks (Kamada et al., 1996). Mkc1 is also important for fungal pathogenesis, as loss of *MKC1* reduces fungal virulence in the mouse systemic infection model (Diez-Orejas et al., 1997). Individually, neither a *mkk2*Δ/Δ nor a *mkc1*Δ/Δ mutation induces changes in -glucan exposure in yeast cells (Roman et al., 2015; Chen et al., 2019a; Correia et al., 2019). However, Mkc1 does appear to impact (1,3)-glucan exposure levels when *de novo* phospholipid biosynthesis is interrupted by loss of the phosphatidylserine synthase gene *CHO1*. A *cho1*Δ/Δ shows increased activation of Mkc1, and disruption of this MAPK in *cho1*Δ*/*Δ (*cho1*Δ*/*Δ *mkc1*Δ*/*Δ double mutant) leads to a further increase of (1,3)-glucan exposure when compared to its already unmasked *cho1*Δ*/*Δ parent strain (Chen et al., 2019a). Thus, it appears that Mkc1 may, in fact, play a role in minimizing unmasking induced by the *cho1*Δ*/*Δ mutation by restoring cell wall integrity. Finally, a hyperactive GTP-bound form of Rho1 (*RHO1*^*Q*67*L*^), located upstream of Mkc1, displays increased (1,3)-glucan exposure compared to wild-type. However, it also exhibits increased phosphorylation of Cek1 along with Mkc1 (Chen et al., 2019a). This suggests that components within the signal transduction pathway can also directly impact unmasking, but it is unclear if the increased -glucan exposure from the hyperactive *RHO1*^*Q*67*L*^ mutant is caused by upregulation of Mkc1 or Cek1, which is a known inducer of unmasking (Chen et al., 2019a,b; Wagner et al., 2021).

## Relevance of ß-Glucan Exposure to Pathogenesis and Disease Control

This review has extensively highlighted environmental signals that either increase or decrease cell wall immunogen exposure, with particular focus on the core cell wall polysaccharide ß(1,3)-glucan, and how *C. albicans* actively perceives these signals to induce cell wall changes. The immunomodulatory impact that these changes have on the immune response has been highlighted as well. However, while this review has focused on individual stimuli that regulate epitope exposure, often the host environment is an amalgamation of host stressors that must be simultaneously accounted for in order to successfully induce disease. For example, hypoxia encountered during disease progression is also often associated with inflammation processes (Grahl et al., 2012), and thus a large number of host immune cells are also actively present within these environments. As previously discussed, NET formation *in vitro* is sufficient to induce unmasking (Hopke et al., 2016), and it is presumed that this also occurs during *in vivo* infection. Yet, the hypoxic environment in which this response is occurring is capable of initiating a response pathway to reduce (1,3)-glucan exposure (Pradhan et al., 2018). Furthermore, neutrophils within a hypoxic/anoxic environment also actively produce and release large amounts of lactate *in vitro*, and this has been proposed to work synergistically with the hypoxic response to further induce masking in *C. albicans* (Lopes et al., 2018). Thus, one can easily envision the arms race between fungal pathogen and the host to influence -glucan exposure levels to favor a given side, and highlights the interplay that these systems likely have during disease progression *in vivo*. Yet, this fine balance provides an attractive target to leverage for disease control in order to provide a favorable outcome for the host.

Concealing immunogenic epitopes, and specifically ß(1,3)-glucan, is a conserved strategy utilized by pathogenic fungi during disease progression, and as such the disruption of these processes may prove useful as a broad targeting approach for fungal disease control. In addition to *C. albicans*, in *Histoplasma capsulatum*, α-glucan and the glycosylhydrolyase Eng1 are important for minimizing β-glucan exposure (Rappleye et al., 2007; Garfoot et al., 2016), and in *Aspergillus fumigatus* biosynthesis of galactosaminogalactan by Uge3 masks hyphal β-glucan from immune detection (Gravelat et al., 2013). In both *C. albicans* and *H. capsulatum*, mutants have been identified that induce unmasking without strongly changing the overall fitness of the fungal pathogen *in vivo*, and attenuate virulence in a manner that depends on a functional host immune response (Garfoot et al., 2016; Wagner et al., 2021). These observations consequently show that inappropriately increasing exposure of immunogenic (1,3)-glucan moieties in the cell wall is indeed an effective strategy to attenuate virulence, and further supports the idea that we can leverage this process as a therapeutic approach. There is also evidence to suggest that the mechanism of clearance for echinocandins *in vivo* also relies on cell wall remodeling and epitope exposure in addition to their fungicidal effects. It has been shown that systemic infection in dectin1^−/−^ mice diminishes the antifungal activities of caspofungin treatment seen in immunocompetent mice (Marakalala et al., 2013). Here, kidney fungal burden was not significantly different in *C. albicans* infected dectin1^−/−^ mice that were given caspofungin or the drug vehicle control, in contrast to wild-type mice where caspofungin did significantly reduce kidney fungal burden. A similar observation was also seen in *C. albicans* infected neutropenic mice receiving anidulafungin treatment (Wiederhold et al., 2012). All the immunocompetent mice that were systemically infected with *C. albicans* and received anidulafungin survived, while ~50–60% of neutropenic mice receiving anidulafungin succumbed to infection. Thus, *in vivo* evidence strongly supports the claim that unmasked fungal cells are more readily recognized and cleared by the host.

Although support exists for utilizing fungal unmasking to facilitate host pathogen clearance, attention must be paid to the infection site for which this strategy is deployed. Many of these studies have been done in systemic infection models, which is only a single manifestation of candidiasis. The observation that loss of the oral epithelial (1,3)-glucan receptor EphA2 further exacerbates oropharyngeal candidiasis in mice suggests that leveraging (1,3)-glucan exposure as an immunotherapeutic approach may be beneficial for fungal clearance within the oral cavity as well (Swidergall et al., 2018). This is also supported by the observation that oral infection with an unmasked *cho1*Δ*/*Δ mutant results in reduced fungal burden on the tongue, although fitness defects caused by loss of *CHO1* also likely contribute to this colonization attenuation (Davis et al., 2018). Similarly, the observation that mutants exhibiting increased (1,3)-glucan unmasking have reduced fitness within the murine gastrointestinal tract also suggests that manipulating this phenotype may work as an effective control strategy within the gut (Sem et al., 2016). Yet, for any infection site, the basis of this approach is dependent on proper recognition and clearance by immune cells. During vulvovaginal candidiasis, neutrophil mediated clearance in the vaginal lumen of mice has been found to be impaired as a consequence of heparan sulfate within the infection site (Yano et al., 2017; Pericolini et al., 2018). The inability to clear fungal cells may therefore result in a situation in which polymorphonuclear cell recruitment is high and immunopathogenesis occurs as a consequence. Indeed, clinical isolates from the vaginal lumen display large amounts of variability in their basal levels of (1,3)-glucan exposure, and a correlation between increased fungal unmasking, neutrophil recruitment and symptom development for the patient has been established (Pericolini et al., 2018; Gerwien et al., 2020). Thus, unlike during systemic and oral infections, leveraging unmasking as a therapeutic approach for vulvovaginal candidiasis may have detrimental outcomes for the host. Therefore, more work is needed to better understand infections in which this therapeutic strategy is appropriate.

## Author Contributions

TC and AW collaborated on the first draft and TR edited it. All authors contributed to the article and approved the submitted version.

## Funding

This was supported by NIH award R01AI153599 (TR) and National Natural Science Foundation of China 82001678 (TC), China Postdoctoral Science Foundation 2020M672065 (TC), and the Fundamental Research Funds of Shandong University 21510072064024 (TC). The funders had no influence on the content of this communication or the decision to publish.

## Conflict of Interest

The authors declare that the research was conducted in the absence of any commercial or financial relationships that could be construed as a potential conflict of interest.

## Publisher's Note

All claims expressed in this article are solely those of the authors and do not necessarily represent those of their affiliated organizations, or those of the publisher, the editors and the reviewers. Any product that may be evaluated in this article, or claim that may be made by its manufacturer, is not guaranteed or endorsed by the publisher.
